# Effect of C-Type Natriuretic Peptide on Maturation and Developmental Competence of Goat Oocytes Matured *In Vitro*

**DOI:** 10.1371/journal.pone.0132318

**Published:** 2015-07-07

**Authors:** Junhong Zhang, Qiang Wei, Jiao Cai, Xiaoe Zhao, Baohua Ma

**Affiliations:** 1 College of Veterinary Medicine, Northwest A&F University, Yangling, Shaanxi, People’s Republic of China; 2 Key Laboratory of Animal Biotechnology, Ministry of Agriculture, Yangling, Shaanxi, People’s Republic of China; China Agricultural University, CHINA

## Abstract

The developmental competence of oocytes during *in vitro* maturation (IVM) is compromised due to asynchronous nuclear and cytoplasmic maturation. To improve IVM efficiency, a pre-maturation culture or two-step maturation strategy has been established, involving meiosis arrest induced by pharmacological agents to provide oocytes with sufficient time to synchronize the maturation of the nucleus and cytoplasm. C-type natriuretic peptide (CNP), which has been demonstrated to function as an oocyte maturation inhibitor (OMI) in many species, provides a new alternative to improve the developmental capacity of oocytes matured *in vitro*. However, the effect of CNP on meiosis arrest and the maturation of goat oocytes remains unclear. In the present study, CNP was shown to function as an OMI in goat oocytes. CNP could temporarily maintain the meiotic arrest of goat oocytes cultured *in vitro* for 4 hours. This transient effect was partly due to the reduction of natriuretic peptide receptor 2 (Npr2). Estradiol could delay the decrease in Npr2 expression and prolong the duration of meiosis arrest up to 6 hours. Based on the above results, a two-step method was established for goat oocyte maturation, in which the oocyte maturation rate was significantly increased. After parthenogenetic activation, the cleavage rate, blastocyst rate and total cell number of blastocysts were significantly improved. Our results suggested that CNP can be used to delay meiotic resumption and enhance the developmental competence of goat oocytes matured *in vitro*.

## Introduction

In mammalian animals, oocytes in antral follicles are arrested at meiotic prophase, known as the germinal vesicle (GV) stage, for a prolonged period [1]. Studies have suggested that oocyte maturation inhibitor (OMI) can be found in follicle fluid and maintains meiotic arrest [2]. Recent studies have indicated that C-type natriuretic peptide (CNP) plays a role as an OMI in many species [3–7]. CNP is produced by follicular mural granulosa cells and is secreted into follicle fluid. Meanwhile, natriuretic peptide receptor 2 (Npr2), the receptor for CNP, is mainly expressed by cumulus cells. The binding of CNP to Npr2 increases the cyclic guanosine monophosphate (cGMP) level in cumulus cells. cGMP diffuses into oocytes through gap junctions and inhibits the activity of phosphodiesterase 3A (PDE3A), resulting in the prevention of cyclic adenosine monophosphate (cAMP) degradation. Elevated cAMP promotes the phosphorylation of cyclin-dependent kinase 1 (CDK1) and inactivates maturation-promoting factor (MPF) [8–9]. In response to the preovulatory luteinizing hormone (LH) surge, meiosis resumes by decreasing the expression of CNP and Npr2 and reducing the guanylyl cyclase activity of Npr2 [10–11].

When immature oocytes are isolated from follicles and cultured *in vitro*, oocytes spontaneously resume meiosis because of escape from the inhibitive environment [12–13]. However, the subsequent developmental competence of oocytes matured *in vitro* is compromised compared with that of their *in vivo* counterparts [14–15]. The poor embryonic developmental capacity is believed to be due to asynchronous nuclear and cytoplasmic maturation [16]. *In vivo*, meiotic arrest provides adequate time for cytoplasmic maturation. Once immature oocytes are isolated and matured *in vitro*, precocious meiosis resumption leads to incomplete cytoplasmic maturation, resulting in a decrease in developmental competence. Therefore, a temporary delay in meiosis resumption during *in vitro* maturation (IVM) could synchronize nuclear and cytoplasmic maturation and improve the developmental competence of IVM oocytes [17–20].

As a physiological meiotic inhibitor existing in follicles, CNP provides a new alternative to synchronize nuclear and cytoplasmic maturation. Although the expression of CNP in granulosa cells of goat follicles has been reported [21], the effect of CNP on meiotic resumption of goat oocytes remains to be determined. In the present study, the effect of CNP on meiotic resumption in goat oocytes was investigated; based on the meiotic inhibitive effect of CNP, we established a “two-step” culture system to improve the developmental competence of goat oocytes matured *in vitro*.

## Materials and Methods

The present study was reviewed and approved by the Institutional Animal Care and Use Committee of College of Veterinary Medicine, Northwest A&F University. All chemicals and reagents were purchased from Sigma (St. Louis, MO, USA)) unless stated otherwise.

### Isolation of goat follicles

Goat ovaries were collected from a local slaughterhouse (Yangling slaughterhouse, Yangling, Shaanxi, People’s Republic of China) and delivered to the laboratory in 0.9% saline solution containing 100 mg/L penicillin and 100 mg/L streptomycin at 22**°C** -25**°C**. Primordial, primary, and secondary follicles were isolated using a mechanical procedure as previously described [22]. After isolation, these follicles were completely removed from the stromal cells and placed by category into separate Eppendorf tubes. All samples were stored at -80**°C** until the RNA was extracted. Third follicles (>2 mm in diameter) visible on the surface of the ovaries were isolated mechanically. Cumulus oocyte complexes (COCs) were punctured out from the third follicles. To collect cumulus cells and denuded oocytes, COCs were repeatedly pipetted. Denuded oocytes or cumulus cells were pooled into individual tubes and stored at -80**°C** until RNA extraction.

### Isolation and culture of goat COCs

COCs were isolated by puncturing the 2- to 5-mm-diameter follicles on the surface of the ovaries. The COCs with more than three cumulus cells layers and a uniform ooplasm were selected for subsequent study. The conventional maturation medium consisted of M199 (Life Technologies, Carlsbad, CA) supplemented with 10% fetal bovine serum (Life Technologies), 1 mM pyruvate, 2.5 mM L-Glutamine, 1% ITS (6.25 ng/mL insulin, 6.25 ng/mL transferrin and 6.25 ng/mL selenium) (Life Technologies), 10 ng/mL EGF, 0.075 IU/mL human menopausal gonadotropin (Serono Laboratories Inc.), and 1 μg/mL 17β-Estradiol. To induce meiosis arrest, COCs were cultured in prematuration medium (first step), consisting of M199 supplemented with 10% fetal bovine serum, 1 mM pyruvate, 2.5 mM L-Glutamine, 1% ITS, different concentrations of CNP (0, 50, 100 and 150 ng/mL) and/or different concentrations of 17β-Estradiol (0, 5, 10 and 50 nM). After induced meiosis arrest, COCs were transferred to conventional maturation medium to complete oocyte maturation (second step).

### Meiotic progrssion assessment

Oocytes were fixed in 4% paraformaldehyde in phosphate buffer saline (PBS) (pH 7.4) for 30 min at room temperature. The nuclei were stained with 10 μg/mL Hoechst 33342. Samples were mounted and observed under an Olympus microscope. According to the observed nuclear state, the nuclei were classified into germinal vesicle (GV), germinal vesicle breakdown (GVBD) and metaphase II (M II) stages (S1 Fig).

### Parthenogenetic activation and embryo culture

Oocytes matured *in vitro* with the first polar body were selected for parthenogenetic activation. The oocytes were exposed to 5 μM ionomycin in mSOFaa for 5 min at room temperature followed by culture in mSOFaa containing 2 mM 6-DMAP for 4 hours. After activation, oocytes were washed completely and cultured in G1.1 medium at 38.5**°C** in a humidified atmosphere of 5% CO2 in air (Day 0). The cleavage rate was recorded after culture for 24 hours (Day 1), and the blastocyst rate and total cell number of blastocysts were subsequently assessed on Day 7 after culture.

### RNA extraction and reverse transcription-polymerase chain reaction

Total RNA was extracted using the TRIzol method. First-strand cDNA was synthesized according to the manufacturer's instructions (Takara, Dalian, China). Specific primers for Npr2 and β-actin were designed using Primer 5.0 software based on the gene sequences in NCBI. The sequences were as follows: Npr2 (accactggcattgtcatggactct and tccttgatgtcacggacgatttcc); β-actin (gtctggcttgagaagaagtagg and gtggagatgaagggaaaagg). The products sizes were 102 bp and 224 bp, respectively.

### Real-time quantitative PCR

The specific primers for real-time PCR and product sizes are the same as mentioned in the above. Real-time PCR was performed using an ABI stepone plus Real-time Detection System (AB, CA, USA) using SYBR Green qPCR SuperMix (Life Technologies). The program consisted of 45 cycles of denaturation at 94**°C** for 5 s, annealing at 60**°C** for 20 s, and extension at 72**°C** for 15 s, with additional extension at 72**°C** for 5 min. Each experiment was repeated independently at least three times, and the fold change in the expression of each gene was analyzed via the 2−ΔΔCt method [23].

### Statistical analysis

Data were analyzed by one-way analysis of variance (ANOVA) using SPSS 13.0 software (SPSS, Inc.). All experiments were repeated at least three times. Variations between replicates are indicated with the standard deviation (±SD in tables and error bars on graphs). Values with a P value less than 0.05 were considered to be statistically significant.

## Results

### Expression of Npr2 in goat follicles

**Fig 1:**
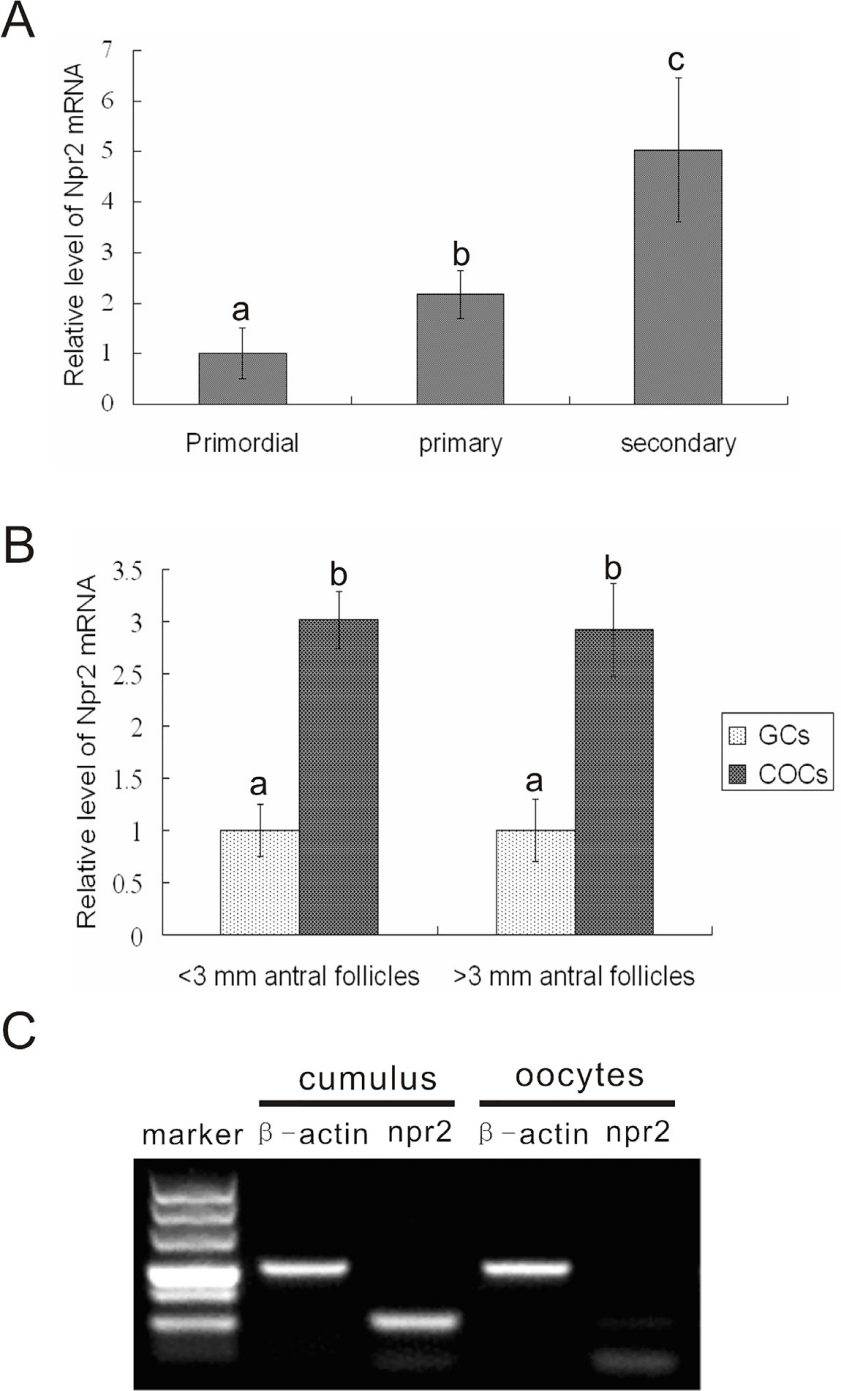
The expression of Npr2 mRNA in goat follicles and COCs. A, The relative levels of Npr2 mRNA in goat preantral follicles at different developmental stages. B, The relative levels of Npr2 mRNA in mural granulosa cells and COCs in goat antral follicles. C, The expression of Npr2 mRNA in cumulus cells and oocytes of COCs in goat antral follicles. Npr2: natriuretic peptide receptor 2; COCs: cumulus oocyte complexes; GCs: granulosa cells.

Because the meiotic arrest function of CNP depends on its receptor Npr2, the expression of Npr2 mRNA in ovarian follicles at different developmental stages was determined. Before antrum formation, the expression of Npr2 mRNA was increased gradually from primordial to secondary follicles (Fig 1A). After antrum formation, the expression levels of Npr2 mRNA was about one-third as high in COCs compared with that in mural granulosa cells, regardless of follicle diameter (Fig 1B). Furthermore, Npr2 mRNA was mainly expressed in cumulus cells rather than in oocytes (Fig 1C).

### CNP temporarily inhibited meiotic resumption of goat oocytes

**Fig 2:**
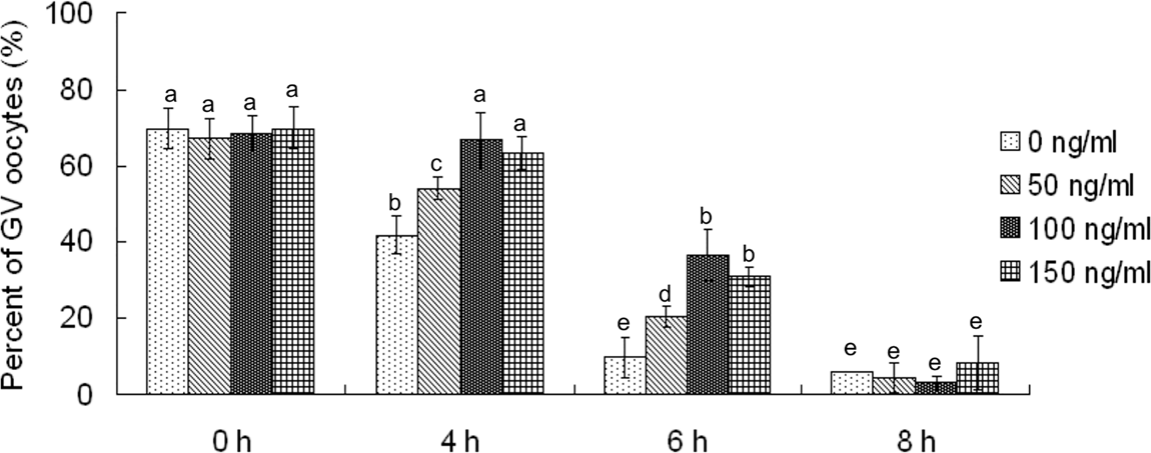
CNP temporarily inhibited the meiotic resumption of goat oocytes cultured *in vitro*. COCs were cultured in medium containing different concentrations (0, 50, 100 and 150 ng/mL) of CNP, and the proportion of oocytes with GV was evaluated after 0, 4, 6, 8 hours of culture, respectively. CNP: C-type natriuretic peptide; COCs: cumulus oocyte complexes; GV: germinal vesicle.

To test the inhibitive effect of CNP on meiotic resumption of goat oocytes, COCs were cultured in medium containing different concentrations (0, 50, 100 and 150 ng/mL) of CNP, and the proportion of oocytes with GV was evaluated after 0, 4, 6, 8 hours of culture, respectively. For COCs freshly isolated from ovaries (0 hours), there were approximately 65% oocytes with GV. After 4 hours of culture, in the 100- and 150-ng/mL CNP groups, the proportions of oocytes with GV did not decrease compared with freshly isolated oocytes (P>0.05) and were significantly higher than those in the 0- and 50-ng/mL CNP groups (P<0.05). After 6 hours of culture, the GV oocyte rates in all of the groups were lower than those in freshly isolated oocytes (P<0.05), although these rates in 100- and 150-ng/mL CNP groups were significantly higher than that those in the 0- and 50-ng/mL CNP groups (P<0.05). Finally, after 8 hours of culture, almost all of the oocytes underwent meiotic resumption in all groups, and there were no significant differences in them (Fig 2). These results suggested that 100- and 150-ng/mL CNP could only temporarily inhibit the meiotic resumption of goat oocytes.

### Effect of estradiol on CNP-induced meiotic arrest of goat oocytes

**Fig 3:**
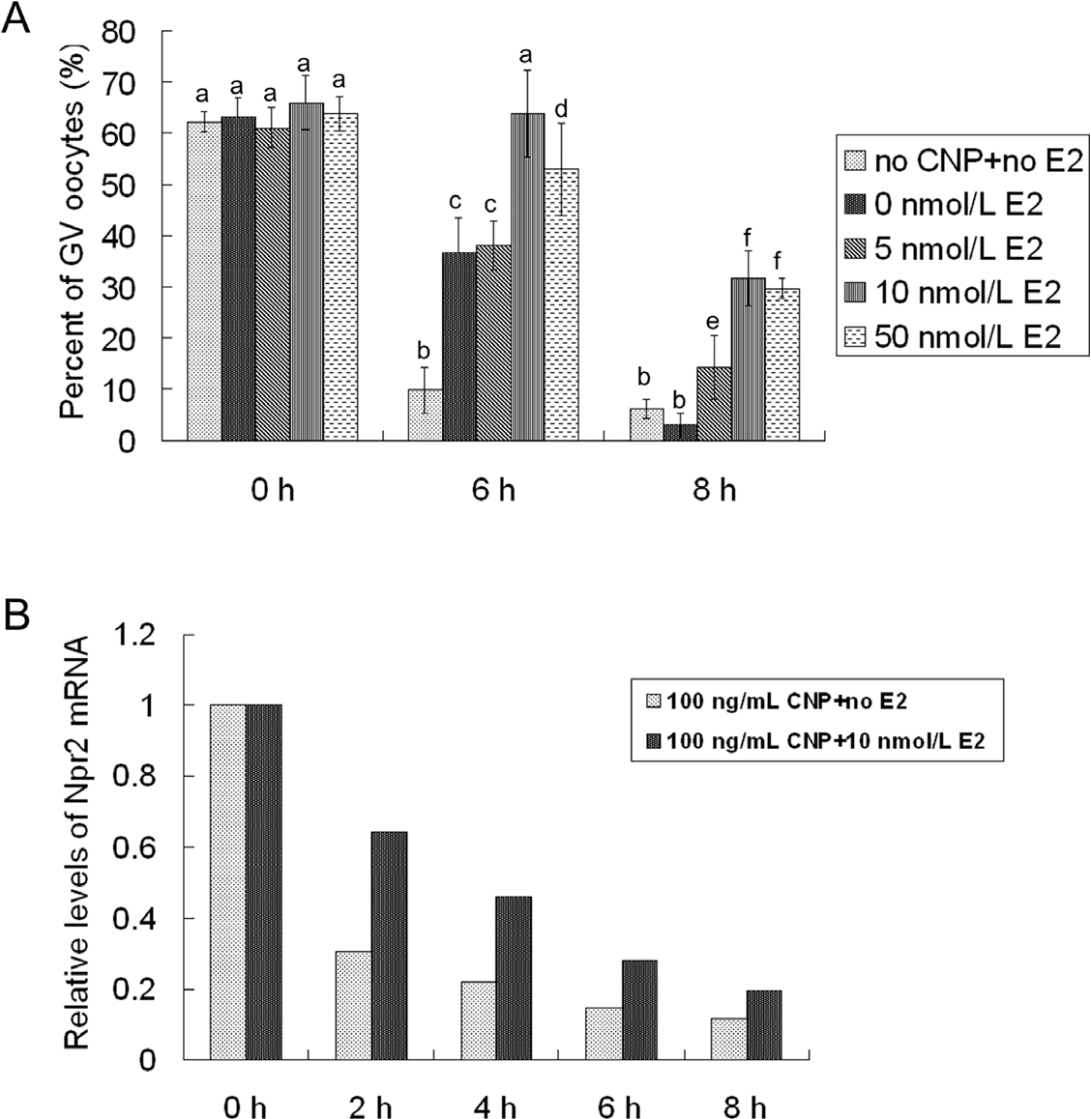
Effect of estradiol on CNP-induced meiotic arrest and the expression of Npr2. A, Effect of estradiol on CNP-induced meiotic arrest. COCs were cultured in medium containing 100 ng/mL CNP and different concentrations (0, 5, 10 and 50 nM) of estradiol. The proportion of oocytes with GV was evaluated after 6 and 8 hours of culture, respectively. B, Effect of estradiol on the expression of Npr2 mRNA. The Npr2 mRNA in COCs cultured in medium containing 100 ng/mL CNP and 10 nM estradiol was detected after 0, 2, 4, 6 and 8 hours of culture, respectively. CNP: C-type natriuretic peptide; Npr2: natriuretic peptide receptor 2; COCs: cumulus oocyte complexes; E2: estradiol.

Because estradiol could maintain the expression of Npr2 in cumulus cells and prolong the duration of CNP-induced meiotic arrest in mouse COCs *in vitro* [24], whether estradiol had the same effect on goat COCs was tested. The goat COCs were cultured in medium containing 100 ng/mL CNP (according to the above result) and different concentrations (0, 5, 10 and 50 nM) of estradiol. After 6 hours of culture, the GV oocyte rate in the 10-nM estradiol group did not decrease compared with freshly isolated oocytes (P>0.05) and was significantly higher than that in the other groups (P<0.05). After 8 hours of culture, however, the GV oocyte rates in all groups were significantly lower than the rate in freshly isolated oocytes (P<0.05), although these rates in the 10- and 50-nM estradiol groups were significantly higher than those in the 0- and 5-nM estradiol groups (P<0.05) (Fig 3A). These results suggested that 10 nmol/L estradiol could prolong the duration of CNP-induced meiotic arrest in goat COCs for at least 2 hours.

### Effect of estradiol on the expression of Npr2 in goat COCs cultured *in vitro*

Spontaneous meiotic resumption is due to the decrease in Npr2 in COCs cultured *in vitro*. The Npr2 mRNA in COCs cultured in medium containing 100 ng/mL CNP and 10 nmol/L estradiol was detected at 0, 2, 4, 6 and 8 hours of culture. As expected, in the absence of estradiol, the expression of Npr2 was acutely decreased during culture. However, estradiol remarkably delayed the decrease of Npr2 (Fig 3B). These results suggested that estradiol could only delay the decrease of Npr2 but not maintain the expression of Npr2 in goat COCs cultured *in vitro*.

### Treatment with CNP during prematuration improved maturation and the developmental competence of goat oocytes matured *in vitro*

Basing on the above results, goat COCs were treated with CNP and estradiol in prematuration medium for 6 hours and then matured in conventional maturation medium for 18 hours (two-steps). COCs matured in conventional maturation medium for 24 hours were used as the control. The maturation rates are summarized in Table 1. In the two-step group, the maturation rate was significantly higher than that in the control group (75.88±5.40% vs 66.41±1.07%, respectively; P<0.05) (Table 1). The mature oocytes were parthenogenetically activated and cultured *in vitro*. As showed in Table 2, the cleavage rate and blastocyst rate in the two-step group were significantly higher than those in the control group (84.24±1.14% vs 72.67±0.50% and 44.80±3.16% vs 30.24±5.60%, respectively; P<0.05) (Table 2). Furthermore, the total cell number of parthenogenetic blastocysts in the two-step group was also higher than that in the control group (149.86±9.26% vs 122.00±10.11%, respectively; P<0.05), and there was no significant difference between the two-step group and *in vivo* group (Table 3).

**Table 1:** Treatment with CNP during prematuration improved the maturation of goat oocytes matured *in vitro*.

Groups	No. of oocytes	No. of maturated oocytes	Maturation rate (%)
Conventional maturation medium	235	156	66.41±1.07^a^
Two-step	248	188	75.88±5.40^b^

*Note*: Values are expressed as means % ± SD.

^a-b^ Different superscripts within the same column are significantly different (P<0.05).

**Table 2:** Treatment with CNP during prematuration improved the developmental competence of goat oocytes matured *in vitro*.

Group	No. of mature oocytes	Cleaved (%)	Blastocyst/Cleaved (%)
conventional maturation medium	154	72.67±0.50^a^(112/154)	30.24±5.60^a^(34/112)
Two-step	147	84.24±1.14^b^(124/147)	44.80±3.16^b^(55/124)

*Note*: Values are expressed as means % ± SD.

^a-b^ Different superscripts within the same column are significantly different (P<0.05).

**Table 3:** Total cell number of blastocysts.

Group	Blastocyst numbers	Total blastomere number
conventional maturation medium	15	122.00±10.11^a^
Two-step	15	149.86±9.26 ^b^
*In vivo*	10	161.20±8.16^b^

*Note*: Values are expressed as means % ± SD.

^a-b^ Different superscripts within the same column are significantly different (P<0.05).

## Discussion

In goat oocytes, the expression of Npr2 mRNA was increased gradually from preantral follicles to antral follicles. Moreover, after antrum formation, the Npr2 mRNA was mainly expressed in cumulus cells rather than in mural granulosa cells and oocytes. These results were consistent with an earlier study in the mouse [3, 25]. Additionally, the expression of Npr2 mRNA in developing follicles indicated the role of CNP in promoting the development of goat follicles.

Although the CNP/Npr2 signaling pathway is essential for meiotic arrest in many other species, its role in the goat has remained unknown. The present study showed that CNP inhibited the meiosis resumption of goat oocytes, supporting that the role of CNP in meiosis arrest was conserved between mammalian species. In the present study, the efficiency of meiotic arrest was improved along with the CNP concentration increasing from 0 to 100 ng/mL. Additionally, only in the 100-ng/mL CNP group, the GV rate was comparable to freshly isolated oocytes. However, the meiotic arrest effect of CNP on goat oocytes was only transient. The GV oocyte rates were distinctly decreased after 6 hours of culture, and most of the oocytes underwent GVBD after 8 hours of culture. Even increasing the CNP concentration over 100 ng/mL did not further prolong the duration of meiotic arrest. Similar to that in the goat, transient meiotic arrest effects of CNP were reported in the murine [3], porcine [5] and bovine [6] species. This phenomenon could be partly explained by the reduction in the expression of Npr2 during culture (Fig 3B). To maintain the expression of Npr2 and prolong the duration of meiosis arrest, estradiol was added to the medium. Unlike in the mouse, in which estradiol could maintain the expression of Npr2 and prolong the duration of meiosis arrest up to at least 24 hours [24], the supplementation of estradiol could not maintain the expression of Npr2 in the goat. Nevertheless, 10 nM estradiol could remarkably delay the decrease in Npr2 expression and prolong the duration of meiosis arrest up to 6 hours. It has been reported that there are putative response elements for the estrogen receptor in the human Npr2 promoter [26]; however, information in goats is still lacking. Therefore, another mechanism of meiotic arrest in the goat could not be excluded in the present study.

Based on the above results, a two-step method was established for goat oocyte maturation. Goat COCs were first meiotically arrested by CNP in prematuration medium for 6 hours and then matured in conventional maturation medium for 18 hours. The results showed that the maturation rate was significantly increased compared with that of the control group. After parthenogenetic activation, the cleavage rate, blastocyst rate and total cell number of blastocysts in the two-step group were significantly higher than those in the control group. These results were in line with previous reports that the delay of meiosis resumption induced by PDE inhibitors [17–20] or CNP [6] was successful in increasing the developmental competence of oocytes matured *in vitro*. In the two-step method, oocytes were meiotically arrested for 6 hours, leaving a relatively longer time for cytoplasmic maturation and improving the synchronization of nuclear and cytoplasmic maturation. Additionally, the meiosis arrest effect of CNP depended on maintaining the in oocyte cAMP level, which ameliorated the oxygen consumption and oxidative metabolism of oocytes [27] and assisted in appropriate spindle formation [28]. On the other hand, CNP could sustain the communication between oocytes and somatic cells [6] that was believed to be beneficial for oocyte developmental competence [29–31]. It has been indicated that CNP could improve oocyte maturation by another mechanism besides the delay of meiosis resumption.

In conclusion, CNP could temporarily maintain the meiotic arrest of goat oocytes cultured *in vitro*. This transient effect was partly due to the reduction in Npr2 expression. Estradiol could delay the decrease in Npr2 expression and prolong the duration of meiosis arrest up to 6 hours. A two-step maturation strategy was established for goat oocytes, in which temporary meiotic arrest for 6 hours induced by CNP increased maturation and improved the developmental competence of goat oocytes matured *in vitro*.

## Supporting Information

S1 Fig Nuclear state of goat oocytes cultured *in vitro*. A, Germinal vesicle (GV) stage; B, Germinal vesicle breakdown (GVBD) stage; C, Metaphase II (M II) stage.
(TIF)
