# Supplementary figures and images for: Effect of C-Type Natriuretic Peptide on Maturation and Developmental Competence of Goat Oocytes Matured In Vitro
Source: PLoS One. 2015 Jul 7;10(7):e0132318. doi: 10.1371/journal.pone.0132318 (PMC4511268; doi:10.1371/journal.pone.0132318)

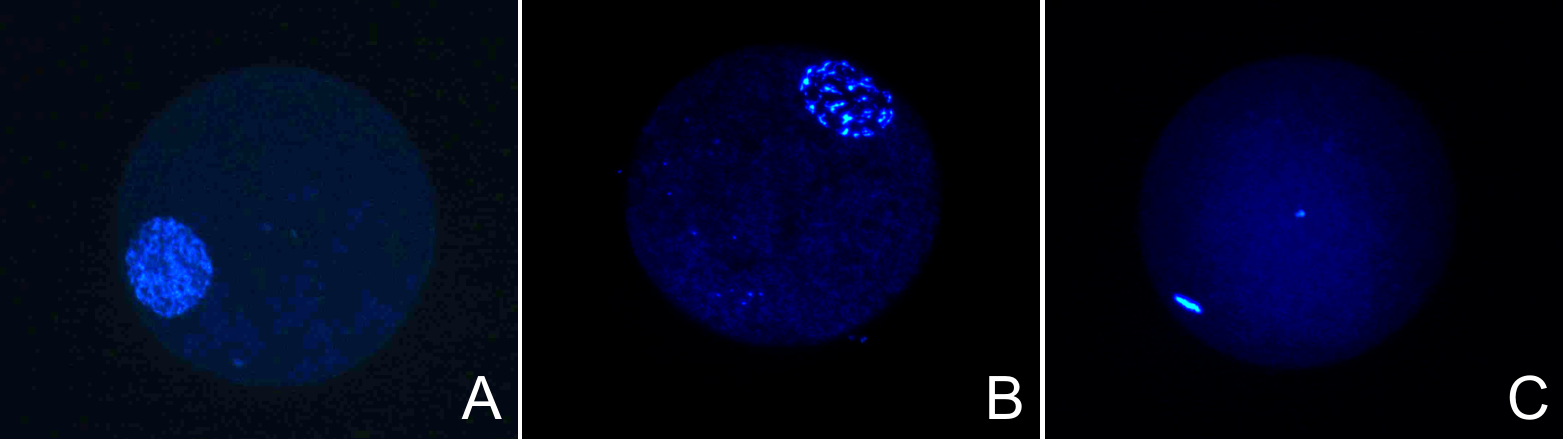

Supplement: S1 Fig — A, Germinal vesicle (GV) stage; B, Germinal vesicle breakdown (GVBD) stage; C, Metaphase II (M II) stage. (TIF) [file pone.0132318.s001.tif]
